# Microbiota and metabolome dynamics induced by Shiga toxin-producing *E. coli* in an *in vitro* model of an infant's colon

**DOI:** 10.15698/mic2025.04.847

**Published:** 2025-04-14

**Authors:** Mariana Izquierdo, Deborah O'Sullivan, Ophélie Uriot, Morgane Brun, Claude Durif, Sylvain Denis, Pablo Gallardo, Cormac G M Gahan, Lucie Etienne-Mesmin, Stéphanie Blanquet-Diot, Mauricio J. Farfan

**Affiliations:** 1 Departamento de Pediatría y Cirugía Infantil Oriente, CICA Hospital Dr. Luis Calvo Mackenna, Facultad de Medicina, Universidad de Chile, 7500539 Santiago, Chile.; 2 UMR 454 INRAe, Microbiology, Digestive Environment and Health (MEDIS), Université Clermont Auvergne, F-63000 Clermont-Ferrand, France.; 3 APC Microbiome Ireland, University College Cork, T12 YT20 Cork, Ireland.; 4 School of Microbiology, University College Cork, T12 K8AF Cork, Ireland.; 5 School of Pharmacy, University College Cork, T12 K8AF Cork, Ireland.; a Equal contribution as a first author.; b Co-last authors.

**Keywords:** Shiga toxin-producing Escherichia coli (STEC), diarrhea, gut microbiota, gut metabolome, Toddler artificial colon model (T-ARCOL)

## Abstract

Shiga toxin-producing Escherichia coli (STEC) is a major food-borne pathogen causing human diseases ranging from diarrhea to life-threatening complications, mainly in young children. Colonization, virulence, and interactions of STEC strains with human gut microbiota are pivotal during infection but remain poorly described, particularly in children, the most affected population. In this work, we evaluated changes in the microbiota and metabolome composition in the *in vitro* gut model: Toddler ARtificial COLon (T-ARCOL) infected with EHEC O157:H7 strain EDL 933. Stool samples collected from children with STEC-positive diarrhea and stool from the same children after recovery from the diarrheal episode (*n=5*) were used to inoculate the T-ARCOL model. STEC colonization was progressively reduced throughout fermentation in T-ARCOL with diarrhea or recovery fecal samples. Beta diversity showed that the diarrhea-associated microbiota was significantly distinct from the recovery microbiota and exhibited a lower α-diversity. In contrast to recovery conditions, diarrheal conditions were characterized by an increased abundance of potential pathobionts such as members of the *Clostridiaceae* family and higher acetate, succinate, and N-acetylneuraminic acid levels. Our results provide new evidence of the impact of EHEC in the microbiota and metabolome dynamics in an in vitro gut model that could be useful in understanding their physiopathology in this at-risk population, considering inter-individual variabilities in gut microbiota.

## Abbreviations

EHEC - Enterohemorrhagic E. coli,

HUS - hemolytic uremic syndrome

SCFAs - short-chain fatty acids,

STEC - Shiga toxin-producing Escherichia coli,

T-ARCOL - Toddler artificial colon model.

## INTRODUCTION

Shiga toxin-producing *Escherichia coli* (STEC) are food-borne pathogens causing acute diarrhea, dysentery, and hemolytic uremic syndrome (HUS). Most outbreaks and sporadic cases of infection worldwide are caused by strains of Enterohemorrhagic *E. coli* (EHEC) serotype O157:H7, a STEC subgroup, transmitted through the ingestion of contaminated food and water 1,2,3,4,5. HUS affects 5-10% of patients, with severity influenced by the bacteria’s genetic virulence profile and the host’s young age 6. Children under the age of five are an at-risk population since diarrheal diseases remain a major cause of infant mortality 7,8. An analysis of fecal microbiota in young children with diarrhea reveals significant alterations in intestinal microbiota composition compared to healthy children, potentially increasing disease risk later in life 9,10,11,12,13.

In addition to Shiga toxins (Stx), which are the main determinants of virulence and major risk factors for severe infections, EHEC strains possess the *eae* gene, encoding for the intimin protein, implicated in attaching and effacing (A/E) lesions to colonize host cells 14,15. Other putative adherence factors, like long polar fimbriae (Lpf), have been identified as contributing to intestinal colonization 16,17,18. EHEC virulence is governed by the Ler and H-NS proteins, which can exert opposing effects on virulence gene expression 19. Virulence gene expression of the pathogen is also strongly regulated by environmental cues, such as temperature, nutrient availability, and pH 17. Mounting evidence suggests that gut microbiota and, in particular, microbiota-derived short-chain fatty acids (SCFAs) might potentially play a pivotal role in the regulation of pathogenic mechanisms and chronic diseases 20,21,22. Apart from differences in gut microbiota composition, analysis of gut metabolome reveals higher SCFA levels in fecal samples of children with diarrhea positive for STEC 13,23. Moreover, different *in vitro* and *in vivo* studies have shown that SCFA may act as signaling molecules potentially influencing virulence functions 24.

Colonization and virulence of STEC strains in the human gastrointestinal tract and their interactions with resident gut microbes are pivotal factors in the infection process 17. However, their characterization remains poorly described, particularly in children, due to the absence of relevant models. Studies in humans are prohibited, and animals do not fully recapitulate the environmental conditions that trigger the expression of virulence genes at infection sites 25,26. Additionally, results obtained from animal models are hampered by differences in digestive physiology and gut microbiota between animals and humans 27,28. *In vitro* human gut models are increasingly considered as an appropriate alternative to *in vivo* assays, provided such systems can accurately replicate physiologically relevant conditions. Roussel *et al*. conducted a comparative study on EHEC O157:H7 survival and virulence under adult and child upper digestive conditions using the dynamic *in vitro* TNO gastrointestinal model (TIM) 29. They demonstrated a significant upregulation of STEC virulence genes (*stx1*, *stx2*, *eae*, *lpf*) under child conditions, suggesting that age-related differences in digestive physicochemical parameters may partly explain the higher susceptibility of young children to EHEC infection and HUS. The group also evaluated the impact of the intestinal ecosystem on the STEC colonization and virulence using the Artificial COLon (ARCOL) model, which reproduces the main nutritional, physicochemical, and microbial conditions of the human adult large intestine 30. In this model, Thévenot et al. showed that EHEC O157:H7 was gradually eliminated from the colon without altering the main populations of resident microbiota 30. These experiments were conducted under adult conditions; and to date no data are available under infant colonic conditions and associated immature microbiota.

In this context, our study aimed to better understand the impact of EHEC O157:H7 on the microbiota and metabolome dynamics in this at-risk population, using the Toddler artificial colon (T-ARCOL) model, recently adapted to mimic the infant colonic ecosystem 31. In a novel approach, we employed multi-omics complimentary techniques to examine the impact of EHEC pathogen on fecal samples used to inoculate the *in vitro* infant colon model, *i.e.*, fecal samples positive for STEC vs. stools collected from the same child after recovery.

## RESULTS

### STEC colonization and virulence gene expression

**Figure 1:**
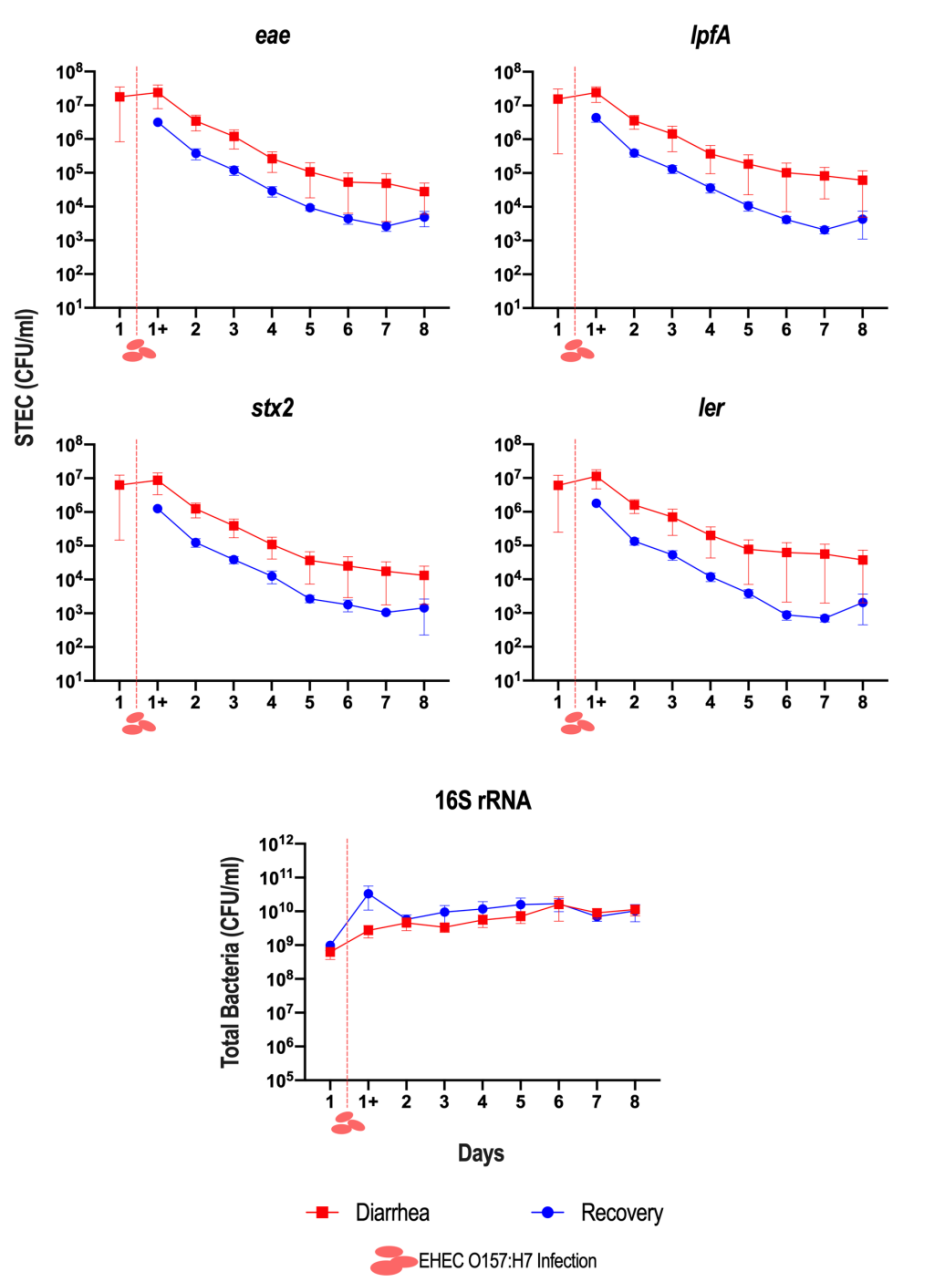
FIGURE 1: Dynamics of STEC colonization in the T-ARCOL inoculated with diarrheal or recovery child fecal samples. Fermentations were run in the T-ARCOL with fecal samples from five children, collected either from the diarrheal episode (red) or the recovery period (blue) and challenged on Day 1 with EHEC O157:H7 strain EDL 933 (dashed red line). Dynamics of STEC colonization were monitored by qPCR targeting genes encoding different virulence factors, namely intimin (*eae*), Ler Regulator (*ler*), Shiga-toxin2 (*stx2*), and long polar fimbriae (*lpfA*). Total bacteria was monitored by the amplification of the 16S rRNA gene. Using a standard curve, cycle threshold (Ct) values were converted into CFU/mL. Results are expressed as mean ± SEM (*n*=5) for each virulence gene. CFU: colony forming unit.

**Figure 2:**
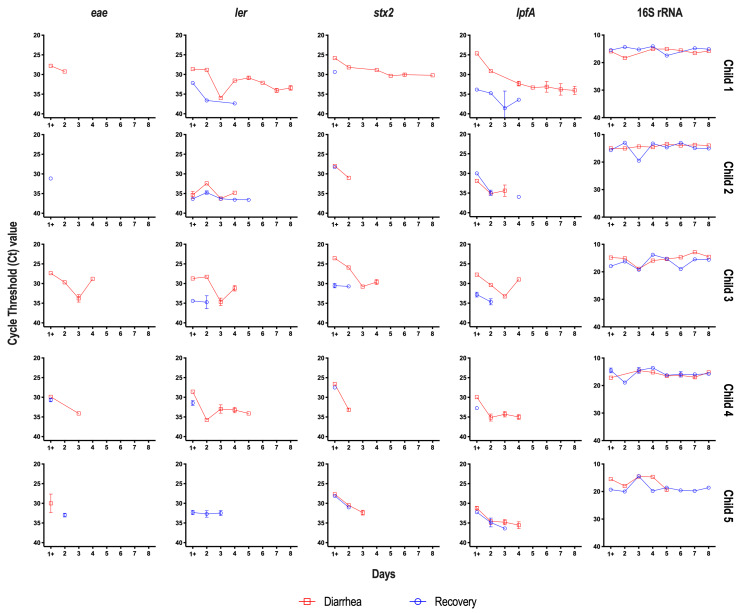
FIGURE 2: Dynamics of STEC virulence gene expression in the T-ARCOL inoculated with diarrheal or recovery stool samples. Fermentations were run in the T-ARCOL with fecal samples from five children, collected either from the diarrheal episode (red) or the recovery period (blue), and challenged on Day 1 with EHEC O157:H7 strain EDL 933. Total bacteria and expression of STEC virulence factors were monitored daily during the total course of fermentation. For STEC virulence, genes encoding intimin (*eae*), Ler Regulator (*ler*), Shiga-toxin2 (*stx2*), and long polar fimbriae (*lpfA*) were monitored, while for total bacteria, the 16S rRNA gene was targeted. Results are expressed as cycle threshold (Ct) value through the fermentation time.

The T-ARCOL was inoculated with diarrhea or recovery samples from children and infected on Day 1 with EHEC O157:H7 EDL 933. STEC colonization during the total course of fermentation was evaluated by qPCR analysis of virulence genes *eae*, *ler*, *stx2*, and *lpfA* (**Figure 1**, Figure S1). In four out of five diarrheal samples, STEC was found in bioreactors (6.1 x 10^6^ ± 1.2 x 10^7^ CFU/mL) on Day 1 of fermentation before infection with EHEC strain EDL 933. STEC colonization was progressively reduced throughout fermentation in the T-ARCOL with the diarrhea or recovery fecal sample. The number of total bacteria, evaluated by targeting the 16S rRNA gene, remained constant from the beginning to the end of fermentation, with no significant difference between the diarrhea and recovery conditions. Additionally, the expression of STEC virulence genes (*eae*, *ler*, *stx*, *lpfA*) was evaluated by RT-qPCR during the total course of *in vitro* fermentations (**Figure 2**). The expression of the *eae* gene was only detected one day following infection with the EHEC strain EDL 933 in all donors, except for Child 3 and Child 4, where *eae* expression persisted until Day 3 and Day 4, respectively, but only in the diarrheal fecal sample. For *ler*, *stx2*, and *lpfA*, gene expression decreased in both diarrheal and recovery conditions, generally remaining undetectable from Day 2 to Day 4, depending on the genes. An exception was observed in Child 1, where these three genes remained expressed with a Ct value in the range of 30-35 until the end of *in vitro* fermentation.

### Gut microbiota diversity

**Figure 3:**
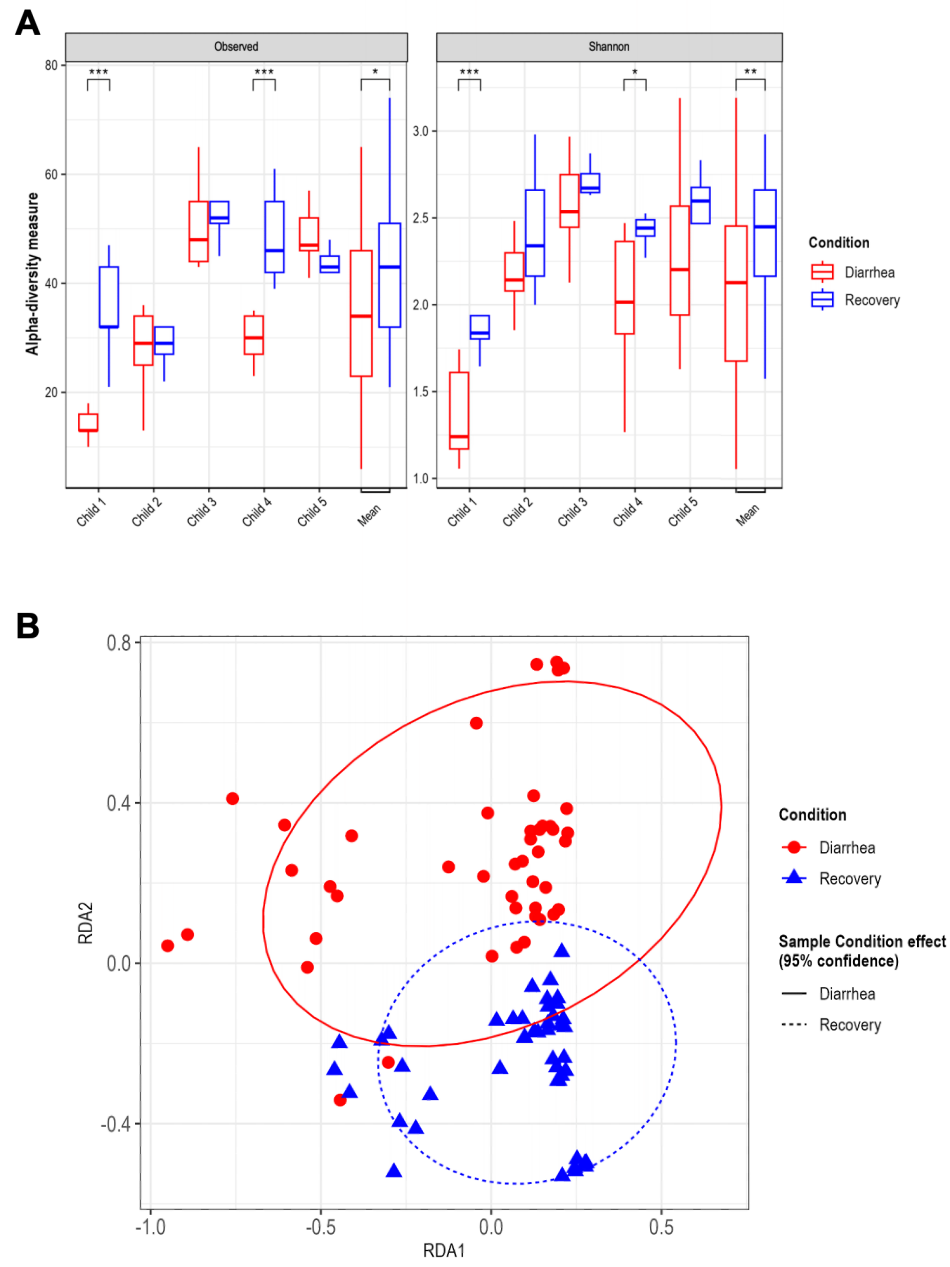
FIGURE 3: Effect of diarrhea-associated microbiota on gut microbial diversity in the T-ARCOL challenged with EHEC O157:H7. Fermentations were run in the T-ARCOL with fecal samples from five children, collected either from the diarrheal episode (red) or the recovery period (blue) and challenged on Day 1 with EHEC O157:H7 strain EDL 933. Samples were regularly collected in the pre- and post-infection periods, and microbiota composition was analyzed using 16S rDNA metabarcoding. **(A)** Alpha diversity in diarrheal and recovery conditions was evaluated by the number of observed ASVs and Shannon index. Results are shown for each child and on average (*n*=5). Significance between groups was assessed with a two-way ANOVA and Wilcoxon pair comparison test (*p<0.05, **p<0.01, and ***p<0.001). **(B)** Bacterial *β*-diversity was represented by a distance-based redundancy analysis (RDA) using the Bray-Curtis matrix, excluding the donor variable. Samples were analyzed considering sample conditions, namely "Diarrheal" vs. "Recovery". ASVs: Amplicon Sequence Variants; RDA: distance-based redundancy analysis.

Colonic microbiota composition during *in vitro* fermentations in the T-ARCOL model was characterized by 16S rDNA metabarcoding after inoculation with diarrheal and recovery fecal samples from the different children. Microbial α-diversity was significantly reduced under diarrheal compared to recovery conditions when data from all donors were pooled. The median [IQR] values were 34 [23-46] vs. 43 [32-51] and 2.13 [1.68-2.45] vs. 2.45 [2.16-2.66] for Observed ASVs and Shannon index, respectively (**Figure 3A**). This difference was particularly striking for Child 1 and Child 4, the youngest donors. In contrast, α-diversity was not impacted by EHEC infection regardless of the donor (Figure S2A). PcoA analysis highlighted that the donor effect had the strongest impact on gut microbiota β-diversity (Figure S3). An RDA analysis removing the donor effect revealed that microbiota composition differed significantly between the type of fecal sample inoculated in the bioreactor (**Figure 3B**, *p* = 0.001). In particular, the diarrheal condition led to a more dispersed β-diversity than the recovery condition, suggesting strong gut microbiota perturbations. Furthermore, the impact of EHEC O157:H7 infection on β-diversity was studied, showing distinct clustering for pre- and post-infection under both the diarrheal and recovery conditions (Figure S2B, *p* < 0.01).

### Gut microbiota composition

**Figure 4:**
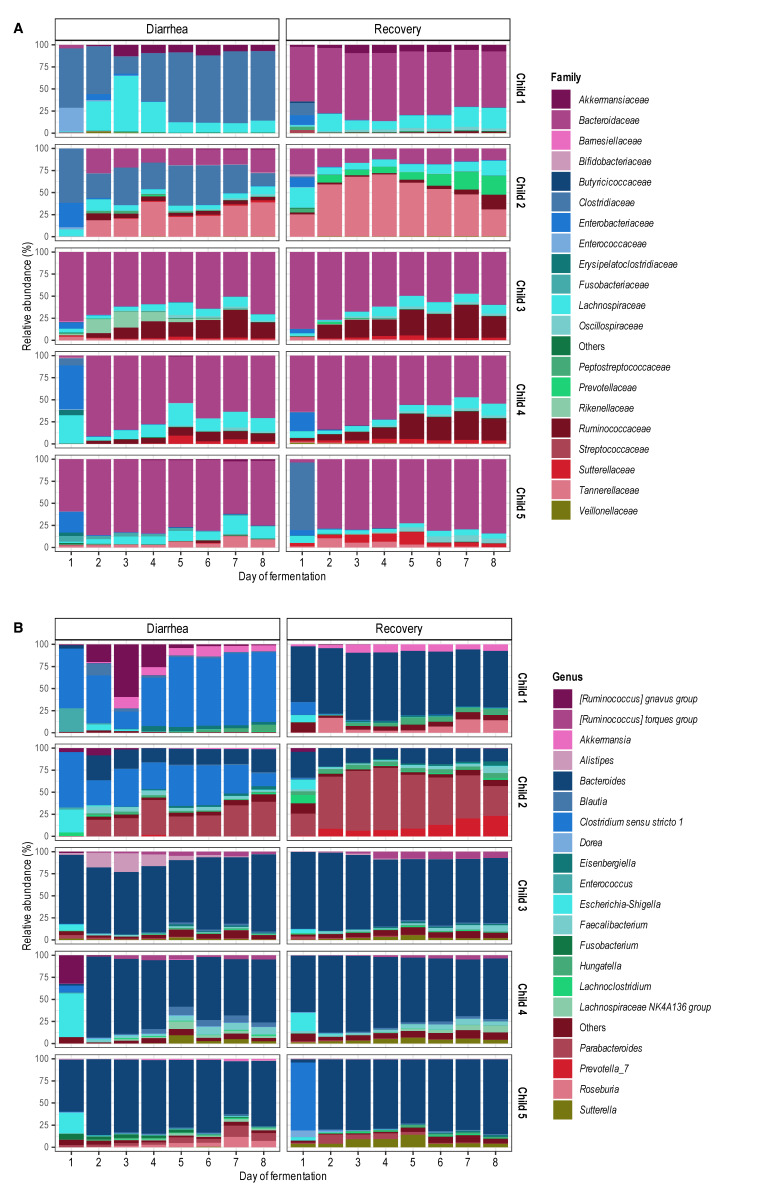
FIGURE 4: Effect of diarrhea-associated microbiota on gut microbiota composition in the T-ARCOL challenged with EHEC O157:H7. Fermentations were run in the T-ARCOL with fecal samples from five children, collected either from the diarrheal episode or the recovery period, and challenged on Day 1 with EHEC O157:H7 strain EDL 933. Samples were regularly collected in the pre- and post-infection periods, and microbiota composition was analyzed using 16S rDNA metabarcoding. Relative abundance of the 20 main bacterial populations at the family (A) and genus (B) levels are shown; less representative taxa are grouped as Others.

The analysis of bacterial abundances at the phylum (Figure S4), family (**Figure 4A**), and genus levels (**Figure 4B**) clearly showed changes in gut microbiota composition between diarrheal and recovery samples (in agreement with **Figure 3**), as well as after adding EHEC O157:H7 to the bioreactors (consistent with Figure S2). At the phylum level, *Bacteroidota* were predominant in all donors, except for Child 1 and Child 2 under diarrheal conditions, where *Firmicutes* were the most abundant. Notably, at the start of fermentation, a relative abundance of *Proteobacteria* ranged from 0 to 50%, which tended to be higher under diarrheal than recovery conditions for most donors. During *in vitro* fermentation in T-ARCOL, the relative abundance of *Proteobacteria* decreased in all donors, regardless of the fecal inoculum, except in Child 1, where *Proteobacteria* increased slightly on Day 2 under diarrheal conditions. Of note, this child is the only one who exhibited up to 10% abundance of *Verrucomicrobacteria* from Day 2-3 under diarrheal and recovery conditions. At the family and genus levels (**Figure 4**), recovery and diarrheal conditions exhibited clearly different bacterial profiles, especially in Child 1 and Child 2. In these donors, members of *Clostridiaceae* family were more abundant in the diarrheal condition compared to the recovery condition, with a higher abundance of the genus *Clostridium sensu stricto*. Under recovery conditions, a bloom in *Prevotellaceae* and *Tannerellaceae* was observed in Child 2. After adding EHEC O157:H7, we observed a different microbiota composition maintained until Day 8 of fermentation with minor fluctuations in relative abundance. Compared to Day 1, in most of the donors, the relative abundance of *Enterobacteriaceae *decreased, and *Bacteroidaceae* increased by Day 2 in diarrheal and recovery conditions. For children 2, 3, and 4, an increase in *Ruminococcaceae *was also found in both fecal samples. An increase in *Akkermansia* was found in both the diarrheal and recovery conditions only in Child 1.

### Gut microbial activities

**Figure 5:**
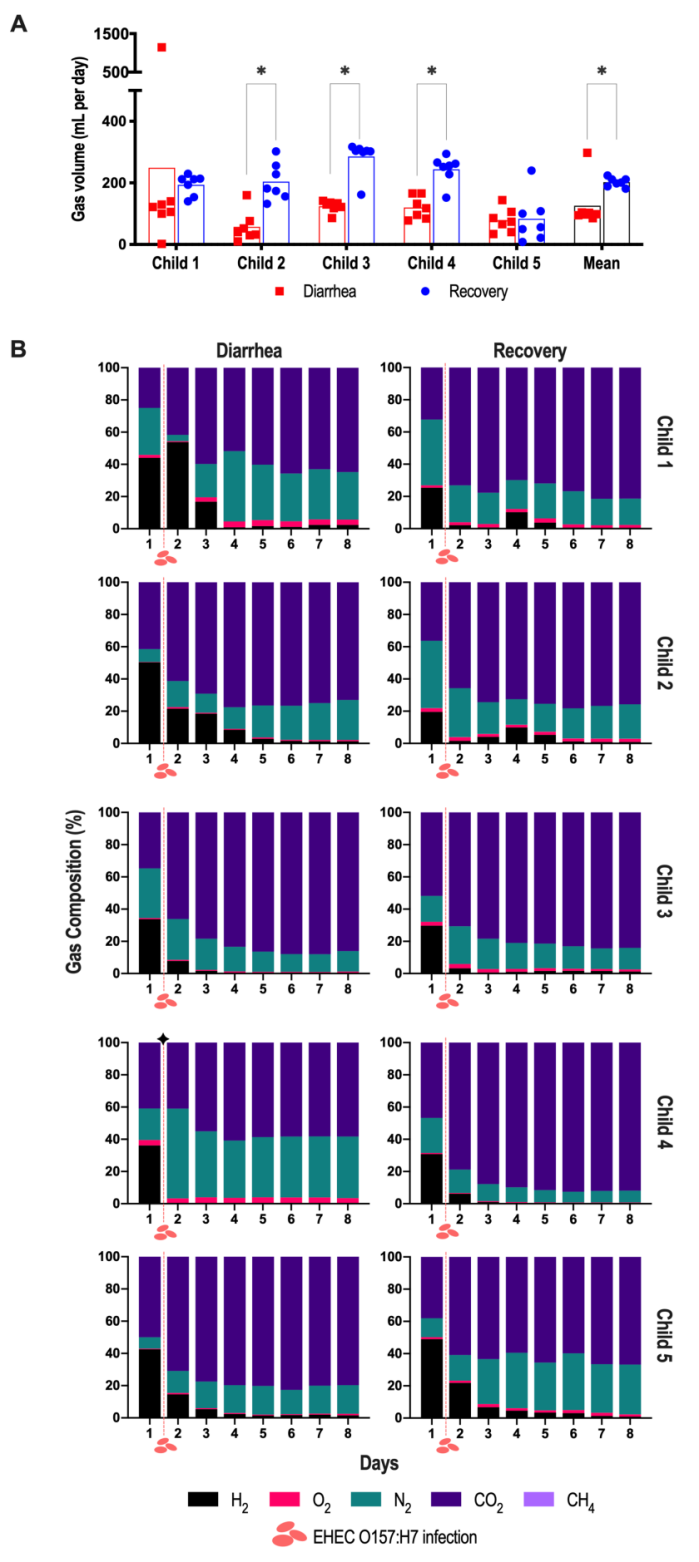
FIGURE 5: Gas production and composition in the atmospheric phases of the T-ARCOL challenged with EHEC O157:H7. Fermentations were run in the T-ARCOL with fecal samples from five children, collected either from the diarrheal episode (red) or the recovery period (blue) and challenged on Day 1 with EHEC O157:H7 strain EDL 933 (dashed red line). Samples were regularly collected in the pre- and post-infection periods in the atmospheric phases of bioreactors. **(A)** Daily gas production (in mL) for each donor and on average (*n*=5). **(B)** Gas composition (in relative percentages) for each donor throughout fermentation. Significance between diarrheal and recovery conditions was assessed with a paired *t*-test (*p<0.05). CH_4_: methane, CO_2_: carbon dioxide, H_2_: dihydrogen, N_2_: nitrogen, O_2_: dioxygen.

In addition to gut microbiota composition and diversity, we also followed microbial activities by sampling in both bioreactors and their atmospheric phases. First, as global indicators of fermentation activities, we measured both the consumption of sodium hydroxide (NaOH) used to maintain the colonic pH at its set point in the T-ARCOL and the redox potential of the fermentation medium (Figure S5). In four out of five children, we found a significantly higher consumption of NaOH under diarrheal compared to recovery condition, with an average of 25.5 vs. 21.9 mL (*n*=5) (Figure S5A). Accordingly, redox potential, which stabilized in the bioreactors around -200 mV, was significantly higher under diarrheal conditions for most of the children (Figure S5B). Regarding atmospheric gasses, we observed a significantly higher daily gas production under recovery compared to diarrheal conditions when all donors were pooled, with 202 vs. 125.8 mL/day; an inter-individual analysis revealed a significantly higher gas production in Children 2, 3, and 4 (**Figure 5A**). Furthermore, gas profiles were individual-dependent and evolved throughout fermentation, with different ratios between the diarrhea and recovery samples (**Figure 5B**). From Day 1 to Day 3, a high proportion of H_2_ (from 20 to 50 %) was found in both diarrheal and recovery samples. Of note, those percentages were higher only in Child 1 and 2 under diarrheal conditions. Then, regardless of the donor and condition, H_2_ percentages decreased with time to disappear, while CO_2_ increased to become predominant. After stabilization, the relative percentages of CO_2_ still differed between the diarrheal and recovery conditions, especially in Child 4 and 5. Finally, it is noteworthy that none of the children exhibited methane production, consistent with their young age.

**Figure 6:**
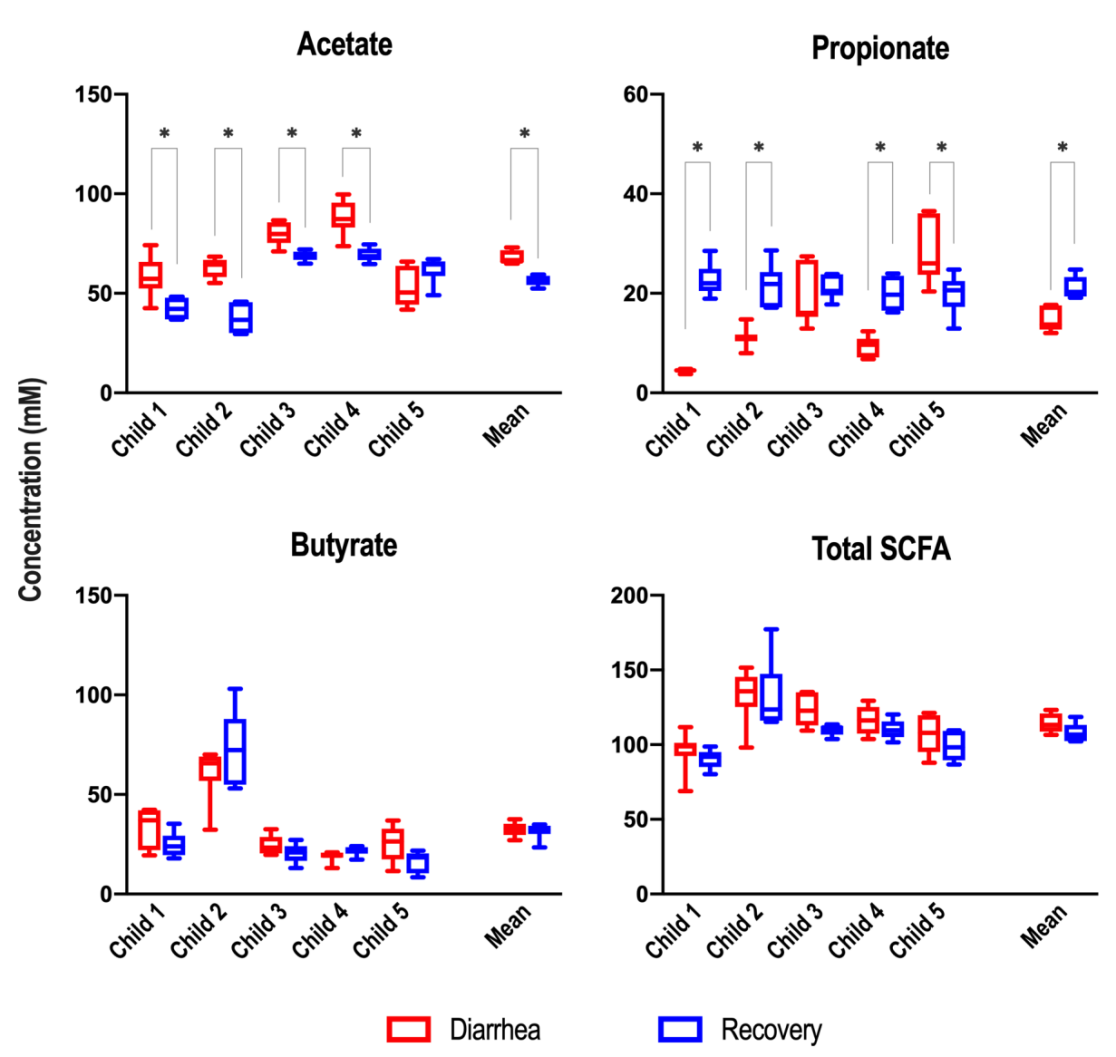
FIGURE 6: Main short-chain fatty acid production in the T-ARCOL challenged with EHEC O157:H7 and inoculated with diarrheal or recovery child fecal samples. Fermentations were run in the T-ARCOL with fecal samples from five children, collected either from the diarrheal episode (red) or the recovery period (blue), and challenged on Day 1 with EHEC O157:H7 strain EDL 933. Daily production of acetate, propionate, butyrate, and total SCFAs (in mM) for each donor and on average (*n*=5) from Day 2 to Day 8. Results are represented as box plots, and the significance between diarrheal and recovery conditions was assessed with a paired t-test (* *p*<0.05). SCFA: short-chain fatty acids.

Other major fermentation end-products, namely the three main SCFAs acetate, butyrate, and propionate were measured in the T-ARCOL (**Figure 6**). No significant difference was found for total SCFA concentration under both conditions. However, when all donors were pooled, significantly higher levels of acetate (68.2 vs. 56.1 mM) and lower levels of propionate (14.4 vs. 21.0 mM) were detected under diarrhea vs. recovery conditions. Inter-individual analysis revealed significantly higher levels of acetate in diarrhea samples in four out of five children (Child 1, 2, 3, and 4), while propionate levels were found to be significantly lower in three children under diarrheal conditions (Child 1, 2, and 4). No significant difference in butyrate concentration between both conditions was observed, regardless of the donor.

### Correlation between the metabolome and microbiota composition

**Figure 7:**
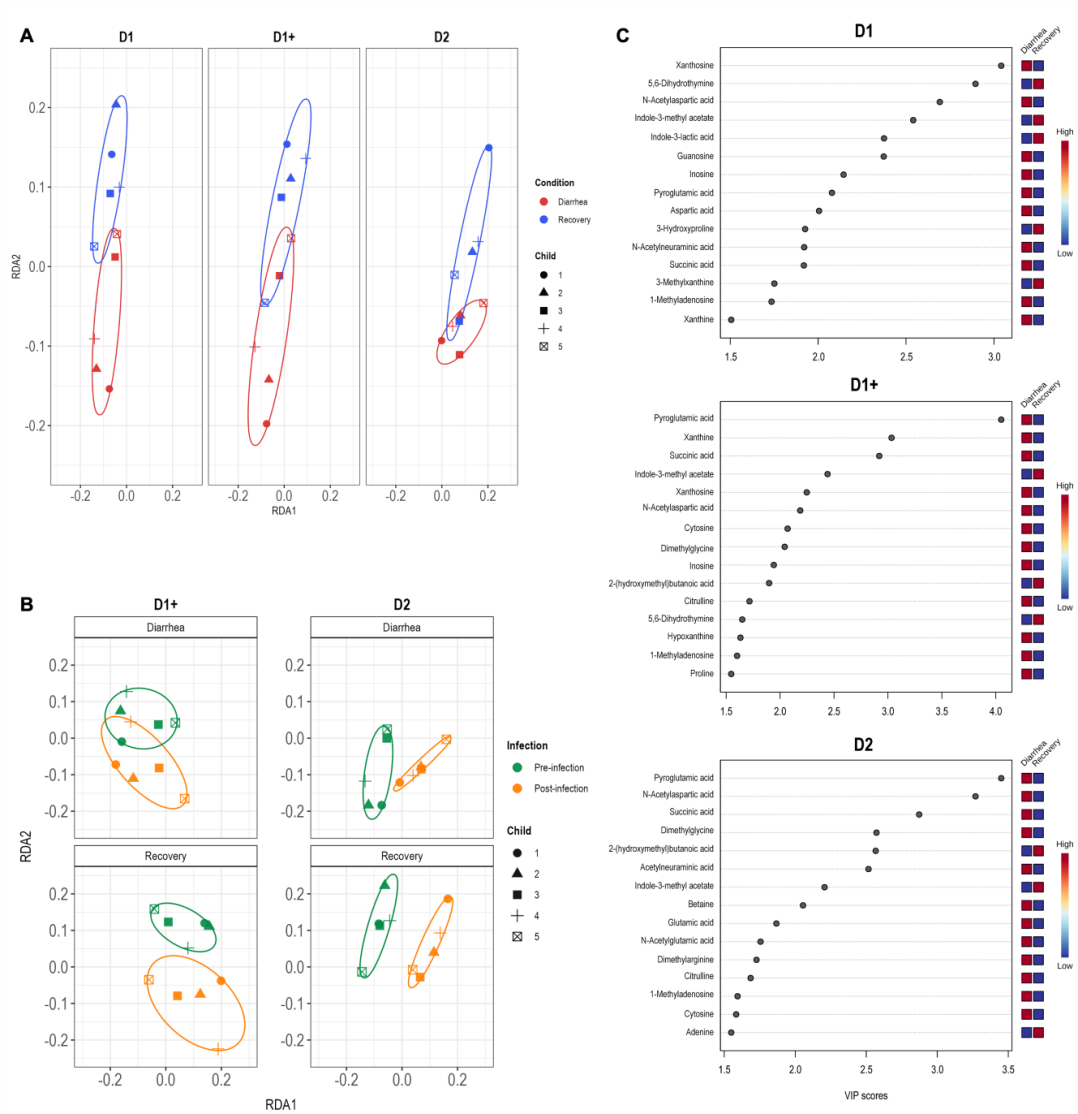
FIGURE 7: Metabolomic analysis of fermentation medium collected from the T-ARCOL challenged with EHEC O157:H7 and inoculated with diarrheal or recovery child fecal samples. Fermentations were run in the T-ARCOL with fecal samples from five children, collected either from the diarrheal episode (red) or the recovery period (blue), and challenged on Day 1 with EHEC O157:H7 strain EDL 933. Samples were collected in the fermentation medium on Days 1, 1+, and 2 for metabolomic analysis. A distance-based redundancy analysis (RDA) using the Bray-Curtis matrix and excluding the donor variable was performed to compare **(A)** "diarrheal" vs. "recovery" conditions at different timepoints and (**B**) "pre-infection" vs. "post-infection" at different timepoints under diarrheal or recovery conditions. **(C)** A partial least squares discriminant analysis (PLS-DA) was performed to identify the main metabolites associated with diarrheal and recovery conditions. Variable importance in projection (VIP) plot displays the top 15 most important metabolite features identified by PLS-DA for each time analyzed. Colored boxes on the right indicate the relative concentration of corresponding metabolites on diarrheal or recovery samples, showing the group with higher or lower concentrations in red and blue, respectively. RDA: distance-based redundancy analysis.

**Figure 8:**
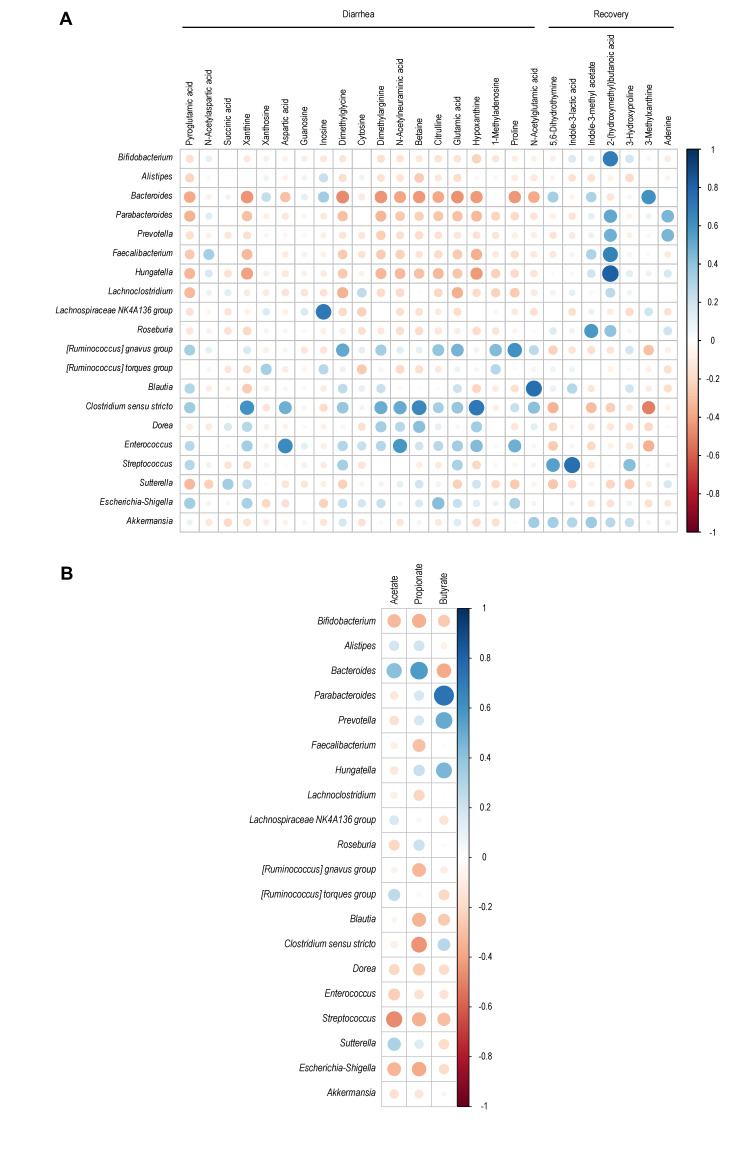
FIGURE 8: Correlation analysis between microbiota composition and metabolites in fermentation samples collected from the T-ARCOL challenged with EHEC O157:H7 and inoculated with diarrheal or recovery child fecal samples. Heatmap showing the correlation relationships between the abundance of the 20 main genera and **(A)** the peak intensity of the 26 metabolites identified by PLS-DA associated with diarrheal and recovery conditions or **(B)** SCFA concentrations. Blue and red dots represent positive and negative correlations, respectively. Color intensity and the size of the circle are proportional to the correlation coefficients. The legend color shows the correlation coefficients and the corresponding colors.

Considering variations in microbiota composition observed on Day 1 (pre-infection), Day 1+ (3 hours post-infection), and Day 2 (post-infection) (**Figure 4**), we analyzed changes in metabolites found in the samples obtained at these timepoints. A total of 124 metabolites were included in the analysis, revealing a distinct metabolome composition between diarrhea and recovery conditions, regardless of the day considered (**Figure 7A**). The effect of infection was also examined, showing significant changes in the metabolome on Day 2 but not Day 1+, compared to samples obtained before EHEC O157:H7 inoculation into the bioreactor (**Figure 7B**). A partial least squares discriminant analysis (PLS-DA) allowed us to identify 26 metabolites responsible for the differences observed in the RDA analyses between diarrhea and recovery samples (**Figure 7C**). Of these, 19 metabolites were related to the diarrhea group, while seven were associated with the recovery samples. Notably, the presence of xanthosine, N-acetylaspartic acid, pyroglutamic acid, xanthine, succinic acid, dimethylglycine, and N-acetylneuraminic acid was associated with diarrheal samples with a Variable importance in projection (VIP) score above 2.5. Conversely, the recovery samples were associated with the metabolites 5,6-dihydrothymine, Indole-3-methyl acetate, and 2-(hydroxymethyl)butanoic acid. We also examined the correlation between the relative abundances of the main bacterial genera and these 26 metabolites (**Figure 8A**). Interestingly, metabolites associated with the diarrheal condition were positively correlated with the presence of the genera *Lachnospiraceae*, *Ruminicoccus*, *Blautia*, *Clostridium*, *Dorea*, *Enterococcus*, and *Escherichia-Shigella*. In contrast, metabolites associated with the recovery condition were positively correlated to the genera *Bifidobacterium*, *Bacteroides*, *Parabacteroides*, *Prevotella*, *Faecalibacterium*, *Hungatella*, and *Streptococcus*. A similar correlation analysis was performed for SCFAs (**Figure 8B**), revealing that butyrate concentration was positively correlated to the presence of *Parabacteroides* and *Prevotella*, while acetate and propionate showed a strong positive correlation with the presence of *Bacteroides*.

## DISCUSSION

Diarrhea caused by enteric pathogens is a major global health problem . Each year, an estimated 2-4 billion cases of infectious diarrhea occur, with infants and young children being particularly affected 35,36. Among pathogens causing enteric diseases in humans, STEC bacteria represent a significant public health concern due to their association with large outbreaks and life-threatening complications 37. STEC infections can lead to severe gastrointestinal issues, including diarrhea and HUS, particularly affecting infants due to their immature immune systems and developing gut microbiota. Previous studies have shown that the fecal microbiota profiles of young children with diarrhea differ significantly from healthy children, with a reduction in taxonomic diversity and richness 9,38. Moreover, prospective studies conducted in Chile by Gallardo *et al*. demonstrated that children with diarrhea caused by diarrheagenic *E. coli* exhibited gut microbiota perturbations compared to age-matched healthy children 12,13. Gut microbiota alterations in children with diarrhea were characterized by a loss of diversity and an increased abundance of *Enterobacteriaceae*, which may increase susceptibility to infections and other gastrointestinal disorders later in life 10,11. In this context, we used the *in vitro* colon model ARCOL for the first time within pediatric parameters (6 months-3 years) 31 to investigate the impact of the gut microbiota from a child with STEC-positive diarrhea and from the same child after recovery from this diarrheal episode on EHEC O157:H7 infection. To consider inter-individual variabilities in gut microbes, experiments were replicated using fecal samples from five donors, two girls and three boys, aged 7 to 39 months. The present study holds particular significance because conducting similar investigations through human clinical trials is not feasible due to ethical constraints associated with inducing pathogenic infections in participants 31.

Our study showed that irrespective of the fecal donor or origin (diarrheal or recovery condition), STEC were progressively eliminated throughout the fermentations, as previously demonstrated during *in vitro* studies in the ARCOL model inoculated with fecal samples collected from healthy adult donors 30,37,39. However, in the T-ARCOL model, STEC persisted for longer than in previous data published with adult ARCOL settings 30, which corroborates *in vivo* findings where prolonged shedding could be observed in young children 40. STEC bacteria were particularly persistent under diarrheal conditions in Child 1, the youngest donor. The pathogen elimination is most likely due to the barrier effect of gut microbiota, termed “colonization resistance”. Resident microbiota can prevent the establishment of intestinal pathogens through various mechanisms, e.g., the production of antimicrobial substances or inhibitory molecules, competition for nutrients or ecological niches, and host immune activation 17. The longer persistence of STEC under pediatric conditions may be linked to the immaturity of their digestive tract and associated gut microbes compared to adults, which is successfully reproduced in the ARCOL model, as previously described 31. We also observed in the present work that diarrhea-associated microbiota correlated with a higher STEC colonization than recovery condition. RDA analysis of β-diversity showed that the diarrhea-associated microbiota was significantly distinct from the recovery microbiota and exhibited a lower α-diversity. Our *in vitro* results align with those of several research groups, showing that the fecal microbiota of young children with diarrhea displayed reduced taxonomic diversity and richness compared to healthy children 9,23. Several reports demonstrated that fecal samples from infants with significantly higher levels of *Proteobacteria* (*Enterobacteriaceae*/*E. coli*) were observed during a diarrheal episode 9,34,41. As expected, we also observed a higher abundance of *Enterobacteriaceae* in Child 3, 4, and 5 under diarrheal conditions. In T-ARCOL, diarrhea-associated microbiota was characterized by a disappearance in microbial markers of health, such as *Ruminococcaceae* and *Prevotellaceae*, and an increase in potentially harmful pathobionts, such as *Clostridiaceae* and *Lachnospiraceae* 42,43,44,45. Together, these data suggest that experiencing a diarrheal episode during childhood may increase susceptibility to pathogenic infection and may be related to a higher risk of disease later in life 46,47,48,49. In addition to colonization, regulating virulence factors in the human gut is a key feature in STEC pathogenesis 50. Shiga toxins (Stx) are crucial determinants of STEC virulence and major risk factors for severe infections. However, pathogenesis is not solely dependent on toxin-mediated effects, and a combination of virulence traits appears to be required 17. Four genes mainly involved in the virulence of STEC strains were studied here: (i) *stx* encoding Shiga toxin 25, (ii) *eae* encoding intimin, required from intimate attachment to the host intestinal mucosa 51, (iii) *ler* a transcriptional activator of the LEE 52, and (iv) *lpf* encoding Long Polar Fimbriae, adhesive factors assumed to be involved in adhesion, translocation, and intestinal inflammation 19. In the T-ARCOL model incorporating infant gut microbiota, we demonstrated that the expression of the four STEC virulence genes (*eae*, *ler*, *stx*, *lpfA*) was consistently higher and persisted longer, up to Day 8 post-infection, in the diarrheal condition compared to the recovery one. Of note, this was particularly true in Child 1, the youngest child, and probably linked to the longer persistence of the pathogen in the colonic model. Currently, there is no available data on the expression of STEC virulence factors within the infant gut *in vivo*. *In vitro*, only three previous studies from our lab have investigated STEC virulence throughout the simulated human digestive tract, but exclusively under healthy conditions 29,37,53. Roussel *et al*. found a significant upregulation of STEC virulence genes (*stx1*, *stx2*, *eae*, *lpf*) in child compared to adult digestive conditions, using the gastric and small intestinal TIM-1 system devoid of intestinal microbiota 29. Again, these *in vitro* observations suggest that age-related variations in digestive parameters may contribute to the heightened vulnerability of young children to STEC infection. Thévenot *et al*. showed in the ARCOL model set up to reproduce healthy adult colonic conditions, overexpression of *stx2* and *eae* genes, up to 12 hours and 6 hours post-infection, respectively 53. In the present study, up-regulation of those genes was observed for up to 8 days and 4 days, respectively, under diarrheal conditions, suggesting that both infant and disease conditions may favor STEC virulence.

Main end-products of microbial fermentations such as SCFAs are important determinants of interactions between gut microbiota and enteric pathogens 54,55; however, evidence regarding the role of SCFA in STEC bacteria remains controversial 17,55,56,57,58. Generally, SCFAs enhance STEC colonization by providing a substrate that the pathogen in the human colon can ferment 17,59. This hypothesis is not in line with our *in vitro* results. Indeed, we observed a higher STEC colonization under diarrhea compared to recovery conditions, whereas similar total SCFA concentrations were measured in both cases. However, when analyzing SCFA profiles, we found significantly higher acetate levels and lower propionate levels in the diarrhea vs. recovery condition, while butyrate concentrations were unchanged. These changes are consistent with previous studies that investigated SCFA levels of fecal samples in large cohorts of children infected with STEC compared to healthy children 13,23. In the T-ARCOL model, acetate levels were positively correlated to *Bacteroides*, one of the predominant acetate-producing bacteria in the human gut 43. Literature has shown that acetate can enhance the growth and virulence of the A/E pathogen *Citrobacter rodentium*, which is extensively used as a murine model for human EHEC infection 60. This data aligns with our *in vitro* results since we observed a higher STEC colonization and increased expression of the four virulence genes under diarrheal conditions. Conversely, it has been demonstrated, in mice, that acetate had a protective effect by inhibiting Stx translocation 61,62, a parameter that cannot be followed in our *in vitro* study due to the lack of human intestinal cells within the ARCOL model.

In addition to the main fermentation end-products, metabolomic analysis revealed other metabolites associated with diarrheal samples, notably xanthine, succinate, and N-acetylneuraminic acid (Neu5Ac). Those metabolites have been positively linked in our *in vitro* study to increases in bacterial populations such as *Clostridium*, *Sutterella*, and *Enterococcus*. In ligated rabbit ileal segment, it has been shown that hypoxanthine (precursor of xanthine) does not decrease the number of STEC bacteria but rather exacerbates the infection and increases the amount of Stx2 toxin produced 63. In a mouse model of EHEC infection, *C. rodentium* can sense succinate through the transcriptional regulator catabolite repressor/activator (Cra) to activate the expression of LEE genes involved in adhesion to the intestinal epithelium 64. The human intestinal epithelium is covered by a complex layer of mucus mainly constituted of secreted oligomerized mucins. EHEC strains have an advantage over commensal *E. coli* in producing a zinc metalloprotease StcE that could release sialic acid such as Neu5Ac and further favor their adhesion and colonization 65. Therefore, our results show that some metabolites found in the T-ARCOL fermentation supernatants under diarrheal conditions could regulate the colonic environment to sustain STEC persistence and virulence.

It is important to note that in four of the five samples used in the present study, STEC was detected in the initial diarrheal samples, so the changes in the intestinal microbiota observed after adding the EHEC O157:H7 strain EDL 933 may also be associated with the presence of STEC strain responsible for causing diarrhea in these children. It is likely that antibiotics or other strategies used to eliminate the pathogen causing diarrhea would have caused alterations in the intestinal microbiota, preventing the evaluation of the changes caused by the addition of EHEC O157:H7 strain EDL 933 in the T-ARCOL itself. Despite this limitation, our results indicate that the introduction of EHEC in the bioreactor induces changes in the intestinal microbiota and metabolome, which pave the way for demonstrating the crucial role played by the gut microbiota and/or gut-microbiota-derived metabolites microbiota in STEC pathogenicity.

Overall, our findings substantiate the emerging perspective that in infants, diarrhea-associated microbiota can affect the clinical course of STEC infection by influencing pathogen colonization and virulence through modulation of the bacterial ecosystem or the production of metabolites such as SCFAs. The contribution of gut microbiota-specific bacteria and its related metabolites to STEC virulence regulation might provide mechanistic hypothesis using the T-ARCOL model. Our study, highlights the significant inter-individual variabilities in gut microbiota structure and functionality, showcasing the relevance of this model to capture these differences, especially in an at-risk population of STEC infection. Next, studies could further use the potential of this *in vitro* model to test remediation strategies such as nutritional approaches, prebiotics, or probiotic strains.

## MATERIAL AND METHODS

### Bacterial strain and culture conditions

The reference strain EHEC O157:H7 EDL 933 used in this study was originally isolated from Michigan ground beef associated with the original 1982 outbreak (ATCC 43895). Bacteria were stored at -80°C in Luria-Bertani (LB) medium containing 20% glycerol. Before the T-ARCOL experiments, the EHEC strain was grown overnight in LB broth at 37°C without shaking until the stationary phase.

### Fecal sample collection and treatment

**Figure 9:**
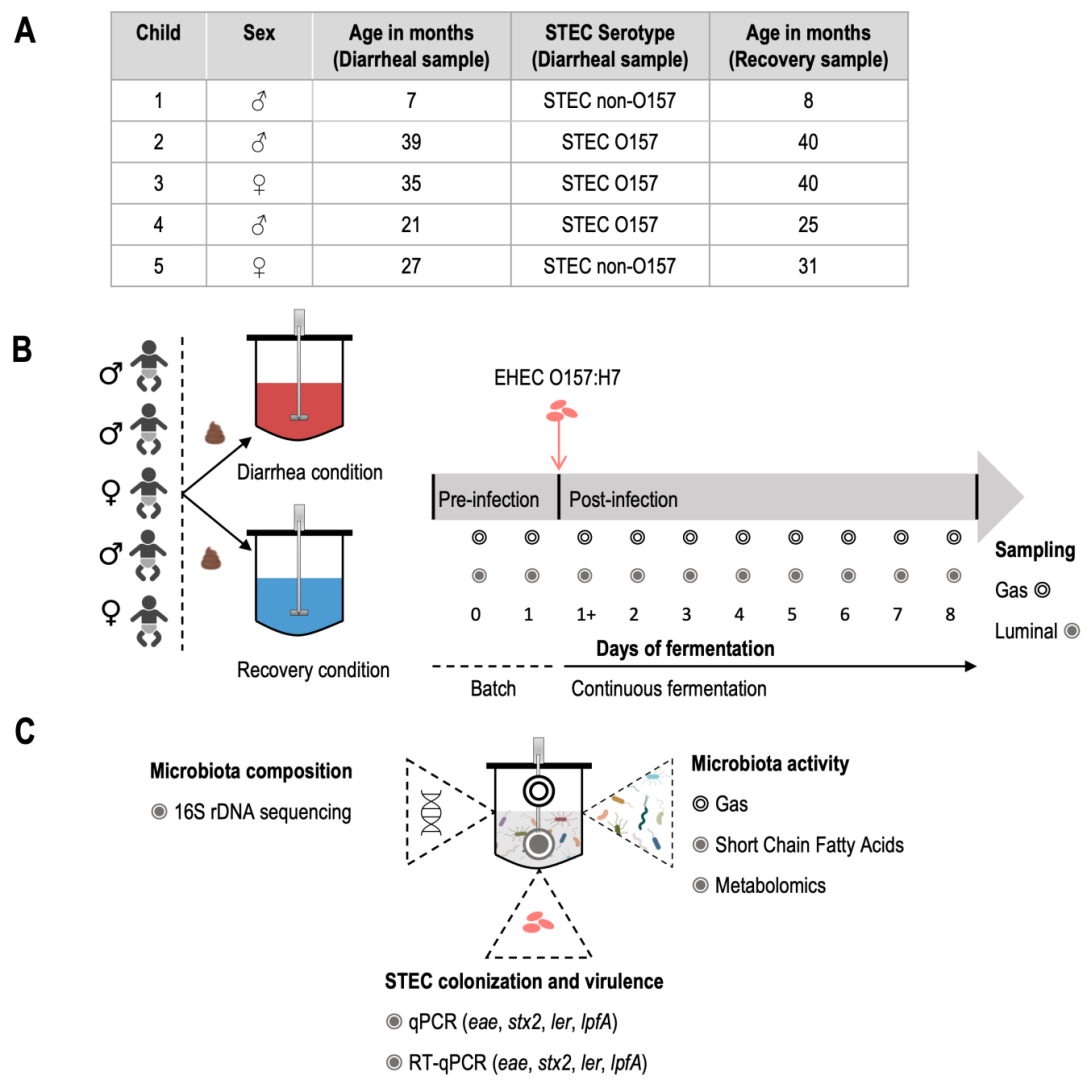
FIGURE 9: Outline of *in vitro* experiments in the T-ARCOL. **(A) **Characteristics of fecal samples collected from five children donors and used to inoculate the bioreactors of the T-ARCOL model (Symbology: female; for female, ♂ for male). **(B)** Two bioreactors from the T-ARCOL model were inoculated with fecal samples from each child (*n*=5) and ran in parallel: one with the fecal sample from STEC-positive diarrhea ("Diarrheal condition") and the other with the fecal sample collected from the same donor after recovery from the diarrheal episode ("Recovery condition"). After an initial batch amplification (24 hours), EHEC O157:H7 strain EDL 933 was introduced in each bioreactor on Day 1. The pre-infection period was defined from Day 0 to Day 1, while the post-infection corresponded to Day 2 to Day 8. Gas and luminal samples were collected daily. An extra sample from the fermentation medium was collected 3 hours after infection (Day 1+). I Samples were used to monitor gut microbiota composition, gut microbial metabolic activities, and EHEC colonization and virulence. qPCR: quantitative PCR, RT-qPCR: Quantitative reverse transcription.

Fecal samples were collected from five children (three boys and two girls, **Figure 9A**) under 3.5 years of age, treated at the Hospital Dr. Luis Calvo Mackenna (HLCM, Santiago, Chile). Two fecal samples were collected from each child: one during a diarrheal episode when the child was positive for STEC and one after the end of the episode when the child no longer presented symptoms. Children did not receive antibiotics, probiotics, steroidal, or nonsteroidal anti-inflammatory drugs two months before enrollment in this study. Written informed consent was obtained from all parents on behalf of their children. The use of fecal samples of human origin was approved by the Universidad de Chile’s Ethics Committee under registration number No. 032-2020. Following defecation, fecal samples were frozen at -80°C without cryoprotectants. All feces were screened for 22 enteric pathogens using FilmArray GI testing (BioFire Diagnostics, USA). For the T-ARCOL experiments, a 5% inoculum (w/v) suspension of fecal samples was prepared for each donor under strict anaerobic conditions (COY Laboratory Products Inc, USA) by mixing feces in sterile sodium phosphate buffer (pH 6.5). The resulting suspension was filtered through a 500-µm sieve in a sterile bottle.

### Experimental design and sampling in the T-ARCOL model

The Toddler Artificial COLon (T-ARCOL) model (Applikon, The Netherlands) is a one-stage fermentation system operated under continuous conditions that reproduces the main nutritional, physicochemical, and microbial parameters of the infant’s colon between 6 months and 3 years based on *in vivo* data 31. At the beginning of the experiment, 100 mL of fecal suspension was added per bioreactor to 200 mL of nutritive medium, reflecting the ileal effluent typical of an infant diet 31. After an initial sparging of O_2_-free N_2_-gas, anaerobiosis was maintained during the total course of the fermentation by the sole activity of the resident microbiota as occurring *in vivo*. Bioreactors were kept at 37°C. Colonic pH and redox potential were continuously recorded (Applikon, The Netherlands), and pH was set at 6.1 and adjusted with the automatic addition of 2 M NaOH. The nutritive medium was continuously introduced into the bioreactors, while the fermentation medium was automatically withdrawn, ensuring the appropriate mean retention time of 19 hours as previously defined 31.

For each experiment, two bioreactors were inoculated in parallel with fecal samples collected from a child with STEC-positive diarrhea (termed "Diarrheal sample") or fecal sample from the same child with no further diarrhea or symptom of infection (termed "Recovery sample"). Following a 24-hour batch period allowing microbial amplification, the two bioreactors were challenged on Day 1 with EHEC O157:H7 strain EDL 933 (1 x 10^7^ colony forming units (CFU)/mL). Following infection, fermentations were conducted for a total of 8 days. Samples from bioreactors were collected 3 hours post-infection (referred to as Day 1+), then every 24 hours and stored at -20°C for SCFA quantification and metabolomic analysis or at -80°C for microbiota characterization and investigation of EHEC colonization and virulence. Additional samples were harvested daily from the atmospheric phases to ascertain anaerobic conditions and determine gas composition. The daily volume of gas produced by microbial activity was also measured using a syringe connected to the sampling bag. The experimental design and sampling of T-ARCOL experiments are summarized in **Figures 9B** and **9C**.

### DNA extraction

Genomic DNA was extracted using the QIAamp Fast DNA Stool Mini Kit (Qiagen, Germany) following the manufacturer’s instructions with minor adjustments, as previously described 66. DNA integrity was verified by agarose gel electrophoresis and Nanodrop 2000 analysis (Thermo Fisher Scientific, USA). The DNA quantity was assessed using the Qubit dsDNA Broad Range Assay Kit (Invitrogen, USA) with a Qubit 2.0 Fluorometer (Invitrogen, USA). Samples were stored at − 20°C before analysis (quantitative PCR and gut microbiota characterization).

### Quantitative PCR

Total bacteria and EHEC colonization in the bioreactors were assessed in the T-ARCOL by quantitative PCR via amplifying the 16S rRNA gene or *stx2*, *eae*, *ler*, and *lpfA* genes, respectively. All primers and probes used in this study are described in Table S1. qPCR analysis was performed on a LightCycler 96 Instrument (Roche, Switzerland) using the TaqMan Universal PCR Master Mix (Applied Biosystems, USA). Each reaction was run in duplicate, with a final volume of 15 µL, in a two-step amplification cycle. The amplification conditions consisted of 1 cycle at 95°C for 10 min, followed by 45 cycles at 95°C for 15 s and 60°C for 60 s. A standard curve was established using a serially diluted culture of the EHEC O157:H7 strain EDL 933 to quantify the concentration of EHEC in the bioreactors. The precise bacterial concentrations were determined in CFU per mL by plating cultures on agar plates. These were subsequently utilized for DNA extraction and qPCR analysis of the aforementioned EHEC genes. Using this standard curve, cycle threshold (Ct) values obtained from the qPCR analysis were converted to bacteria number per milliliter, and EHEC colonization in the bioreactors was expressed as CFU/mL throughout *in vitro* fermentations.

### 16S rDNA metabarcoding analysis of gut microbiota composition

DNA amplification and sequencing of the 16S rRNA gene was performed according to the preparation guide from Illumina (Illumina Technical Note 15044223). Briefly, the V3-V4 region of 16S rDNA was amplified by PCR with the primers suggested by Klindworth *et al*. 67 (Table S1). The amplification conditions consisted of 1 cycle at 95°C for 3 min, followed by 25 cycles of 95°C for 30 s, 55°C for 30 s, and 72°C for 30 s, and a final cycle at 72°C for 5 min. Amplicon PCR was purified using AMPure XP beads. Illumina sequencing adapters and dual-index barcodes (Nextera XT indices) were added to the amplicon target via PCR amplification under the following conditions: 1 cycle at 95°C for 3 min, 8 cycles at 95°C for 30 s, 55°C for 30 s and 72°C for 30 s, and a final cycle at 72°C for 5 min. The 16S rDNA library was sequenced on the Illumina MiSeq platform (HLCM, Santiago, Chile) using a 2 x 300 bp paired-end reagent kit. A bioinformatic analysis was performed following the DADA2 pipeline 68 on R software (version 4.1.2, 2021-11-01). Demultiplexed raw sequences data in fastq format were quality filtered, where reads with N bases were eliminated and reads under 100 bp long were removed. Decontamination steps were performed to filter out sequences corresponding to PhiX DNA, which was used as a spike-in control for MiSeq runs. Filtered sequences were dereplicated and cleaned from chimeras. Amplicon sequence variants (ASVs) were inferred from the remaining sequences using the dada function from the dada2 package. A table with ASVs counts for each sample and representative sequences of ASVs in fasta format were generated. Taxonomic affiliation of all ASVs was performed with the assignTaxonomy function using the SILVA release 138.1 database (80% bootstrap cut-off) as a reference 69. A phylogenetic tree was constructed based on ASVs representative sequences using functions from the phangorn package 70.

### RNA extraction

Samples collected from the T-ARCOL at different timepoints were centrifuged (2000g, 7 min, 4°C) and pellets were resuspended with RNAlater reagent (Invitrogen) and stored at -80°C. Total RNAs were extracted using the TRIzol^™^ reagent (Invitrogen, USA). To remove any contaminating genomic DNA, treatment with DNase I (Qiagen, Germany) was performed as previously described 71. RNA concentrations were evaluated using a Synergy HT spectrophotometer (Biotek, USA).

### Quantitative reverse transcription (RT-qPCR) analysis of EHEC virulence genes

RNA samples were used to analyze the expression of EHEC virulence genes (*eae*, *ler*, *stx2*, and *lpfA*) by RT-qPCR. The expression of the 16S rRNA gene was used as an internal control. cDNA amplification was achieved using TaqMan Fast Virus 1 Step Master Mix (Applied Biosystems, USA), using primers and probes described in Table S1. Each reaction was run in duplicate with a final volume of 15 μL, using a LightCycler 96 Instrument (Roche, Switzerland). The amplification conditions consisted of 1 cycle at 50°C for 5 min, 1 cycle at 95°C for 20 s, followed by 45 cycles at 95°C for 15 s and 60°C for 60 s. Ct values obtained from PCR analysis were used to evaluate EHEC virulence gene expression changes during fermentation.

### Gas composition analysis

To determine the percentage of the gases (H_2_, O_2_, N_2_, CO_2_, and CH_4_) present in the atmospheric phases of T-ARCOL bioreactors, gas samples collected during fermentations were analyzed in an HP 6890 gas chromatograph (Agilent Technologies, Santa Clara, CA, USA) coupled with a thermal conductivity detector (TCD). Two series columns, Molecular Sieve 5A and PoraPlot U, were used. Gas composition was determined using calibration curves made from ambient air (78,09% N_2_, 20,95% O_2_, 0,04% CO_2_) and three gas mixtures A (5% CO_2_, 5% H_2_, 90% N_2_), B (19.98% CO_2_, 80,02% H_2_), and C (19,89% CO_2_, 19,88% CH_4_, 20% H_2_, 40,23% N_2_). Two technical replicates were performed for each sample, and the results were expressed as relative percentages.

### Short-chain fatty acids analysis

Samples collected from bioreactors at different timepoints were centrifuged twice (14000g, 15 min, 4°C, then 16000g, 30 min, 4°C), and supernatants were filtered with a 0.2 μm filter. These supernatants were used to determine the concentration of three main SCFAs (acetate, propionate, and butyrate) using a high-performance liquid chromatography instrument with a UV detector (HPLC-UV 1260 Infinity; Agilent Technologies, USA) at the HLCM (Santiago, Chile) as previously described 23,72. Briefly, samples were chemically extracted, and 20 µL aliquot of each sample was injected into the HPLC-UV instrument. The analyte was separated using a Hypersil Gold aQ column (150mmx4.6mm id) with a particle size of 3 µm (Thermo Scientific, USA). The mobile phase consisted of a mix between 20 mM NaH_2_PO_4_ in HPLC water (pH 2.2) and acetonitrile. The UV detector was set at a wavelength of 210 nm. Peak intensities were analyzed with the Agilent OpenLAB CDS software (Agilent, USA), and concentrations (mM) were determined using a standard curve.

### Metabolite analysis by untargeted metabolomics

Filtered supernatants from bioreactors collected on Day 1, Day 1+, and Day 2 were sent to MS-Omics company (MS-Omics, Denmark) for metabolomic profiling of semi-polar metabolites using ultra-performance liquid chromatography (UPLC) coupled with high-resolution quadrupole-orbitrap mass spectrometer (Orbitrap Exploris 240 MS, Thermo Fisher Scientific, USA) according to the MS-Omics method. A total of 150 metabolites were detected. A filtering step was performed using KEGG 73 and PubChem 74 databases, selecting 124 metabolites with a bacterial or human origin to continue the analysis.

### Statistical Analysis

Statistical analyses on gut microbiota activity (gas and SCFA) were processed using GraphPad Prism software (version 9). Data normal distribution was verified by combining the Anderson-Darling, D'Agostino & Pearson, Shapiro-Wilk, and Kolmogorov-Smirnov tests. Then, appropriate statistical analysis was applied (one-way ANOVA, *t*-test, or Mann-Whitney test). A *p*-value <0.05 was considered statistically significant. A metabarcoding analysis, including α diversity indices (number of observed ASVs and Shannon index), *β*-diversity, and bacterial relative abundance at the phylum, family, and genus levels, was performed using the R software (version 4.1.2). For the *α*- and *β*-diversity analysis, unweighted and weighted Unifrac distance matrices were generated, and a redundancy analysis (RDA) ordination was used for visualization. A principal coordinate analysis (PCoA) was performed, followed by non-metric multidimensional scaling (NMDS), highlighting important donor and condition (i.e., diarrheal and recovery) effects. A constrained RDA was then performed with time, treatment, and infection as variables of the model and with removal of the donor effect. Bray Curtis distances were used for each analysis, and the enclosing ellipses were drawn based on a 95% confidence interval or Khachiyan’s algorithm. Significance between groups was assessed with an ANOVA permutation test for RDA. Metabolomic analyses were also processed using R Software (version 4.1.2), where peak intensity data were normalized by log transformation, without other data scaling, and used as the input data. RDA was done using the vegan package 75, following the same parameters described for microbiota analysis. The main metabolites associated with each group were identified using a partial least squares discriminant analysis (PLS-DA) and represented by a variable importance in projection (VIP) plot using the MetaboAnalystR package 76. Finally, a correlation analysis between microbiota and metabolites or SCFA was performed using the corrplot package 77 for R Software. The relative abundance of 20 main genera was used for this analysis, along with peak intensity values of metabolites identified by PLS-DA analysis and concentration values for acetate, propionate, and butyrate.

### Data Availability Statement

The 16S rDNA metabarcoding data were deposited and are publicly available in the NCBI Sequence Read Archive (SRA) database with accession number PRJNA1134643.

### Institutional Review Board Statement

The Ethics Committee of the Universidad de Chile approved the study under registration number No. 032-2020 concerning the use of fecal samples of human origin.

### Informed Consent Statement

Informed consent was obtained from all subjects involved in the study before stool collection.

## CONFLICT OF INTEREST

The authors declare no conflicts of interest. The funders had no role in the study’s design, in the data collection, analyses, or inter-pretation, in the writing of the manuscript, or in the decision to pub-lish the results.

## SUPPLEMENTAL MATERIAL

Click here for supplemental data file.

All supplemental data for this article are available online at www.microbialcell.com/researcharticles/2025a-izquierdo-microbial-cell/.
