# Supplementary material for: Microbiota and metabolome dynamics induced by Shiga toxin-producing E. coli in an in vitro model of an infant's colon
Source: Microb Cell. 2025 Apr 14;12:76–92. doi: 10.15698/mic2025.04.847 (PMC12042126; doi:10.15698/mic2025.04.847)
Supplement: Supplementary file 1 [file mic-12-076-s01.pdf]

Supplemental Data

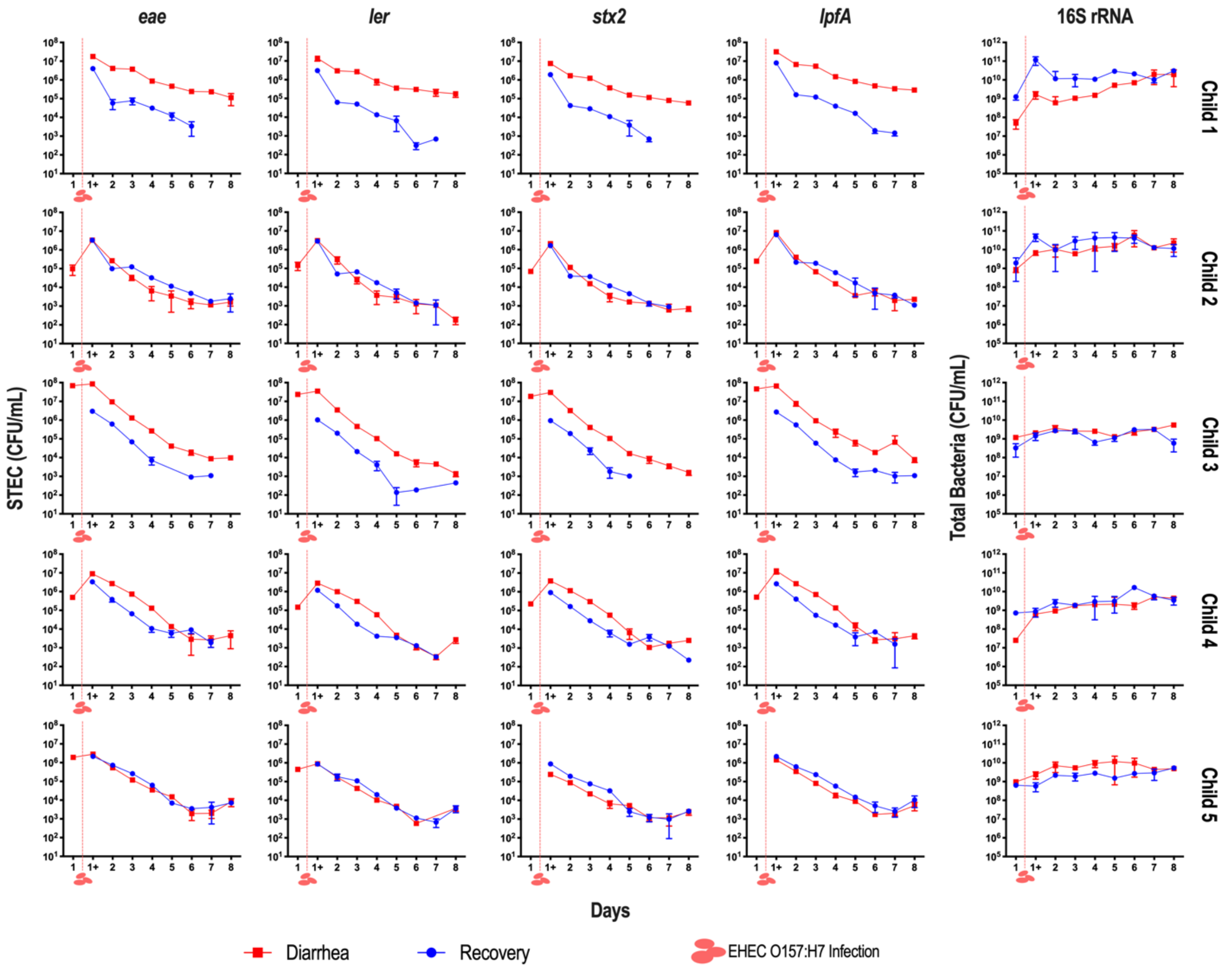

Figure S1. Individual donor dynamics of STEC colonization in the T-ARCOL inoculated with diarrheal or recovery stool samples.

Fermentations were run in the T-ARCOL with fecal samples from 5 children, collected either from the diarrheal episode (red) or the recovery period (blue) and challenged at Day 1 with EHEC O157:H7 strain EDL 933 (dashed red line). The dynamics of STEC colonization were monitored for each child by qPCR targeting genes encoding different virulence factors, namely intimin (*eae*), Ler Regulator (*ler*), Shiga-toxin2 (*stx2*) and long polar fimbriae (*lpfA*). Total bacteria were monitored by the amplification of the 16S rRNA gene. Using a standard curve, cycle threshold (Ct) values were converted into colony-forming units per mL (CFU/mL).

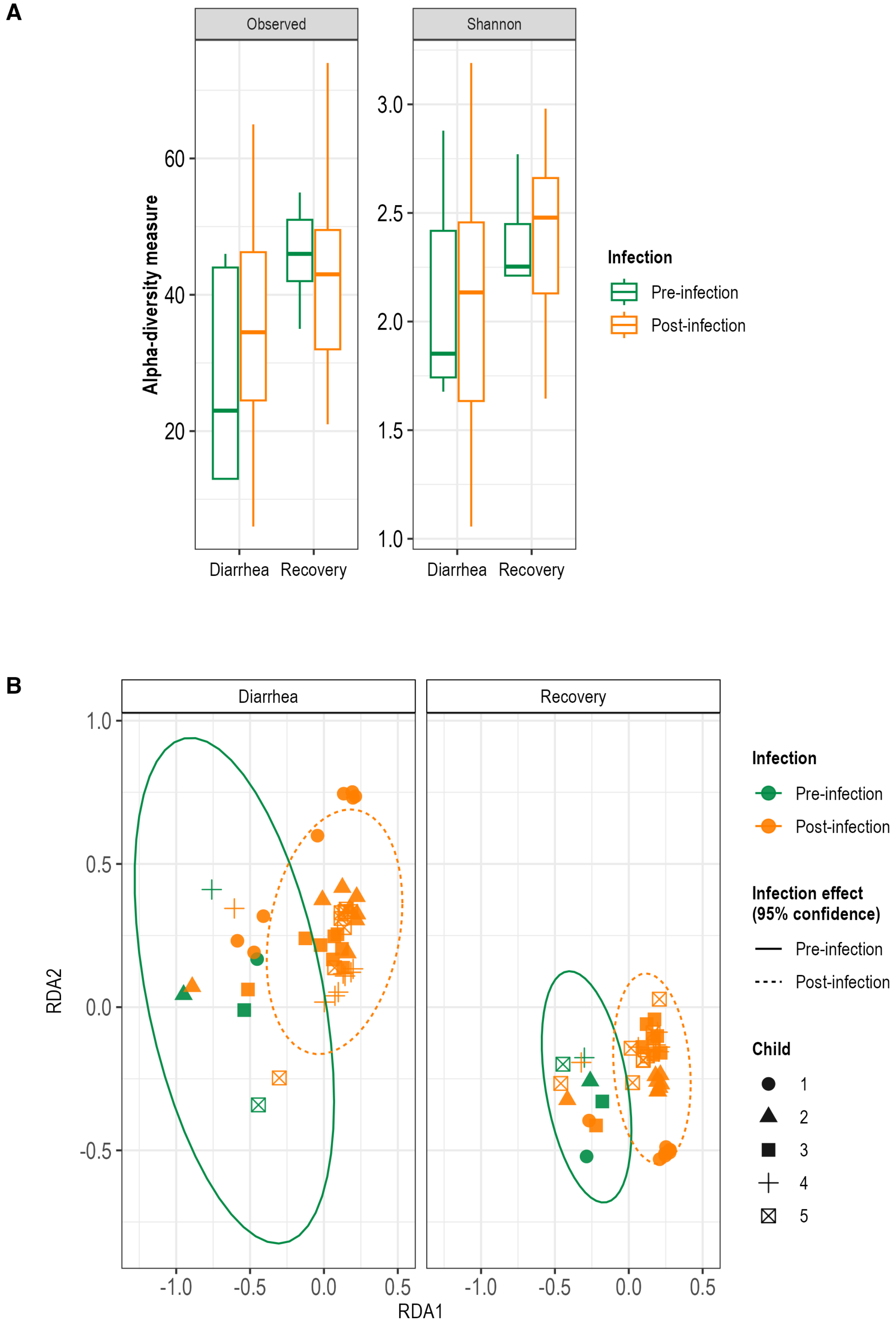
A
Observed
Shannon
Alpha-diversity measure
60
40
20
3.0
2.5
2.0
1.5
1.0
Diarrhea
Recovery
Diarrhea
Recovery
Infection
Pre-infection
Post-infection
B
Diarrhea
Recovery
1.0
0.5
0.0
-0.5
RDA2
-1.0
-0.5
0.0
0.5
-1.0
-0.5
0.0
0.5
RDA1
Infection
Pre-infection
Post-infection
Infection effect
(95% confidence)
Pre-infection
Post-infection
Child
1
2
3
4
5

Figure S2. Effect of challenge with EHEC O157:H7 on bacterial diversity in the T-ARCOL

Fermentations were run in the T-ARCOL with fecal samples from 5 children, collected either from the diarrheal episode or the recovery period, and challenged at Day 1 with EHEC O157:H7 strain EDL 933. Samples were regularly collected in the pre- and post-infection periods, and microbiota composition was analyzed using 16S rDNA metabarcoding. (**A**) The number of observed ASVs evaluated alpha diversity and Shannon indices in each child's pre- and post-infections. (**B**) Bacterial β diversity was represented by a distance-based redundancy analysis (RDA) using the Bray-Curtis matrix, excluding the donor variable. Samples were clustered by infection periods, with ‘pre-infection’ in green and ‘post-infection’ in orange (95% confidence ellipses).

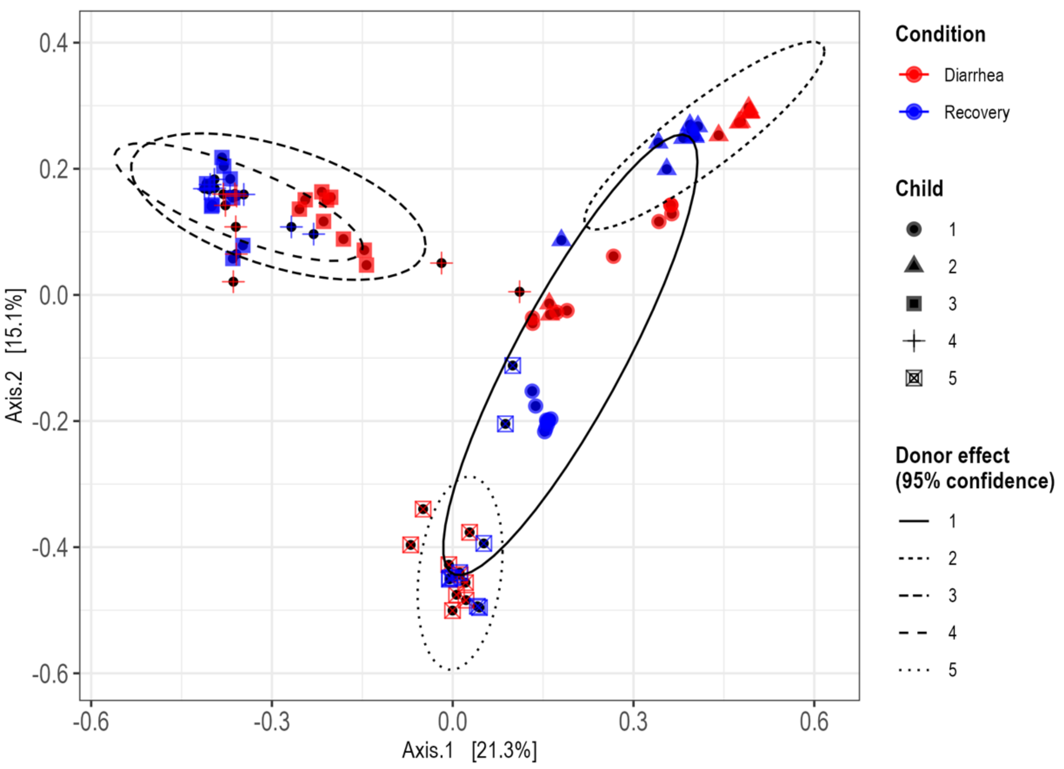

Figure S3. Donor effect on bacterial diversity in the T-ARCOL challenged with EHEC O157:H7

Fermentations were run in the T-ARCOL with fecal samples from 5 children, collected either from the diarrheal episode or the recovery period, and challenged at Day 1 with EHEC O157:H7 strain EDL 933. Bacterial β diversity was represented by using a two-dimensional PCoA plot. Samples were colored by sample condition (diarrhea in red, recovery in blue) and clustered by donor (95% confidence ellipses).

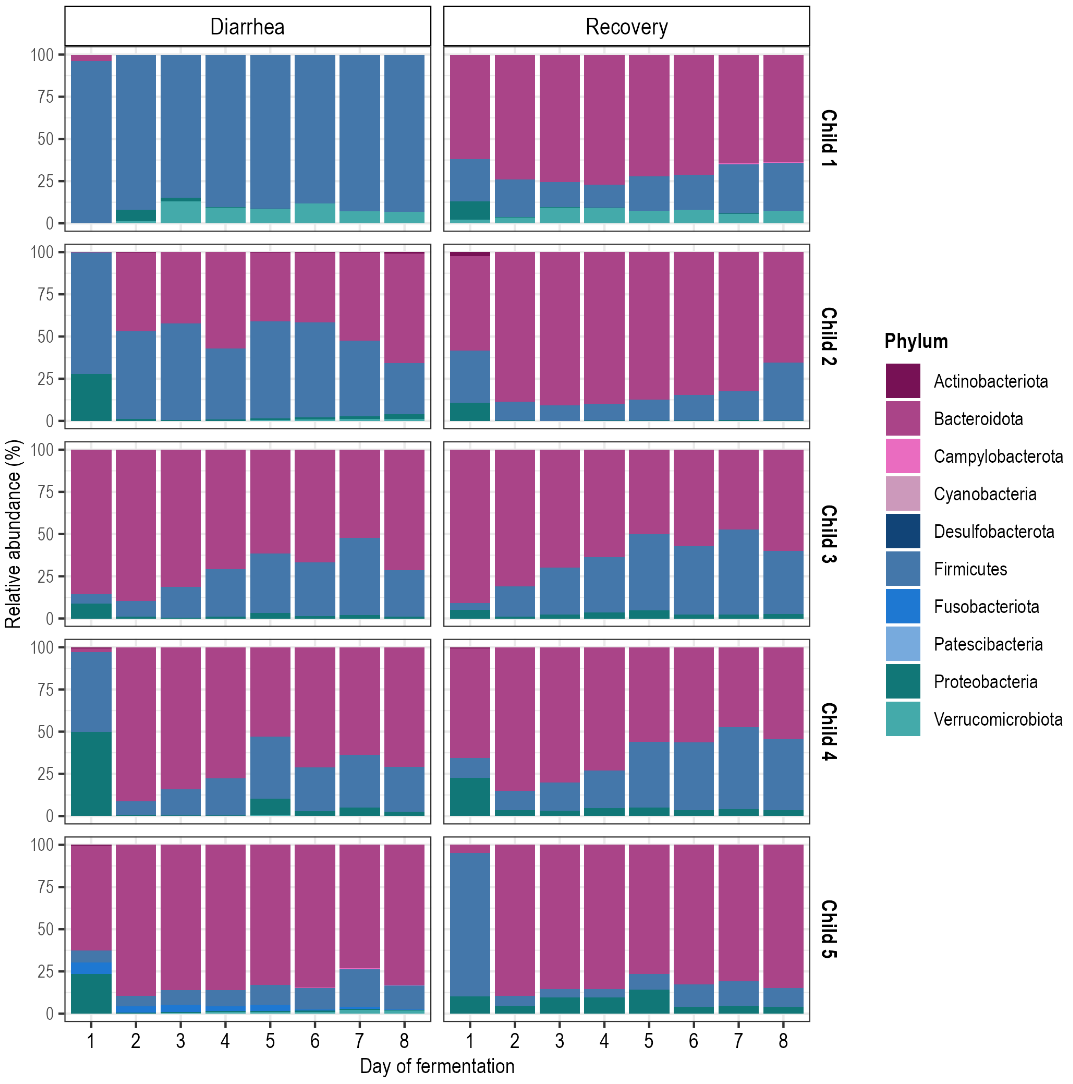

Figure S4. Effect of diarrhea-associated microbiota on gut microbiota composition in the T-ARCOL challenged with EHEC O157:H7

Fermentations were run in the T-ARCOL with fecal samples from 5 children, collected either from the diarrheal episode or the recovery period, and challenged at Day 1 with EHEC O157:H7 strain EDL 933. Samples were regularly collected in the pre- and post-infection periods, and microbiota composition was analyzed using 16S rDNA metabarcoding. The relative abundance of bacterial populations was measured at the phylum level.

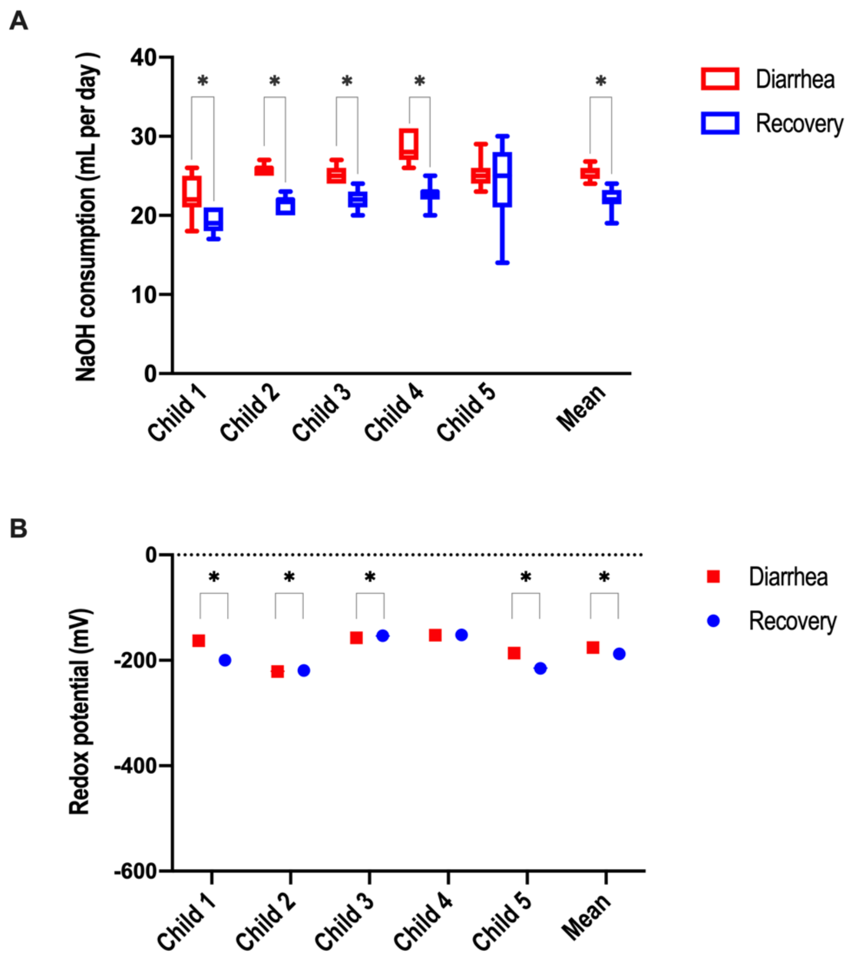

Figure S5. Effect of diarrhea-associated microbiota on microbial activities in the T-ARCOL challenged with EHEC O157:H7

Fermentations were run in the T-ARCOL with fecal samples from 5 children, collected either from the diarrheal episode (red) or the recovery period (blue), and challenged at Day 1 with EHEC O157:H7 strain EDL 933. **(A)** Daily consumption of NaOH delivered within the *in vitro* colonic model to maintain pH at the set point (in mL). Results are represented as box plots for each child and on average (n=5). **(B)** Redox potential recorded throughout fermentation (in mV). Results are expressed as mean ± SEM (n=5). Significance between diarrheal and recovery conditions was assessed with the paired t-test (* P <0.05).

Table S1. Primers and probes used in this study for qPCR, RT-qPCR, and 16S rDNA metabarcoding analysis.

Gene	Target	Sequence	Reference
STEC virulence genes analysis			
eae	Intimin	F: 5’ - CATTGATCAGGATTTTTCTGGTGATA – 3’<br>R: 5’ - CTCATG CGG AAA TAG CCG TTA – 3’<br>P: 5’ - FAM-ATAGTCTCG/ZEN/CCAGTATTCGCCACCAATACC/3IABkFQ – 3’	[78]
ler	Ler virulence Regulator	F: 5’ - AGTTCTACAGCAGGAAGCAAAG – 3’<br>R: 5’ - GAGTCCATCATCAGGCACATTAG – 3’<br>P: 5’ - FAM-CGACTGCGA/ZEN/GAGCAGGAAGTTCAA/3IABkFQ – 3’	This study
stx2	Shiga Toxin 2	F: 5’ - GCTGGAATCTGCAACCGTTACT – 3’<br>R: 5’ - CACGAATCAGGTTATGCCTCAGT – 3’<br>P: 5’ - FAM-CTGCACTTC/ZEN/AGCAAATCCGGAGCCT/3´IABkFQ – 3’	[79]
lpfA	Subunit A, Long Polar Fimbriae	F: 5’ - CAATACCCTGGTCGCTCTTAAC – 3’<br>R: 5’ - CGTTGCCAGAGTAGAAACGTAG – 3’<br>P: 5’ - FAM-TCTGAGTAA/ZEN/AGGCCAGACGGTTCTCT/3IABkFQ – 3’	This study
**16S rRNA**	16S ribosomal RNA	F: 5’ - AATAAATCATAAACTCCTACGGGAGGCAGCAGT – 3’<br>R: 5’ - AATAAATCATAACCTAGCTATTACCGCGGCTGCT – 3’<br>P: 5’ - FAM-CGGCTAACT/ZEN/MCGTGCCAG/3IABkFQ – 3’	[80]
16S rDNA metabarcoding			
**16S rRNA**	V3-V4 region 16S ribosomal RNA	F: 5’ - CCTACGGGNGGCWGCAG – 3’<br>R: 5’ - GACTACHVGGGTATCTAATCC – 3’	[67]

F: Forward primer; R: Reverse primer; P: Probe
